# Female urinary incontinence and sexuality

**DOI:** 10.1590/S1677-5538.IBJU.2016.0102

**Published:** 2017

**Authors:** Renato Lains Mota

**Affiliations:** 1 Departamento de Urologia, Centro Hospitalar de Lisboa Ocidental, EPE e; Universidade Lusófona de Lisboa, Portugal

**Keywords:** Urinary Incontinence, Sexuality, Quality of Life

## Abstract

Urinary incontinence is a common problem among women and it is estimated that between 15 and 55% of them complain of lower urinary symptoms. The most prevalent form of urinary incontinence is associated with stress, followed by mixed urinary incontinence and urge urinary incontinence. It is a symptom with several effects on quality of life of women mainly in their social, familiar and sexual domains. Female reproductive and urinary systems share anatomical structures, which promotes that urinary problems interfere with sexual function in females.

This article is a review of both the concepts of female urinary incontinence and its impact on global and sexual quality of life. Nowadays, it is assumed that urinary incontinence, especially urge urinary incontinence, promotes anxiety and several self-esteem damages in women. The odour and the fear of incontinence during sexual intercourse affect female sexual function and this is related with the unpredictability and the chronicity of incontinence, namely urge urinary incontinence.

Female urinary incontinence management involves conservative (pelvic floor muscle training), surgical and pharmacological treatment. Both conservative and surgical treatments have been studied about its benefit in urinary incontinence and also the impact among female sexual function. Unfortunately, there are sparse articles that evaluate the benefits of female sexual function with drug management of incontinence.

## INTRODUCTION

Urinary incontinence refers to any involuntary leakage of urine with social and hygienic distress, according to the classification of International Continence Society and International Urogynecological Association. It affects men and women, with higher prevalence in women, affecting 15 to 55% (1). In Portugal, according to published data by Portuguese Urological, Neurological and Urogynecological Associations, it is prevalent in 20% of women older than 40 years old, with higher incidence in older women (2). It is an important public health issue due to high prevalence and impact on quality of life (3) and due to financial costs of treatment (4). Clinical investigation of pelvic floor alterations domain generated recommendations and practical guidelines recognized by several international societies of urology and gynecology (5-7). However, they poorly address sexual dysfunctions related to urinary incontinence (although with an evident association, due to anatomic proximity of reproductive and urinary systems) and the impact of sexuality in quality of life and general satisfaction of women (8).

### Investigation methodology

A data search was performed at PubMed using the following filters: “sexual function” OR “sexuality” and “urinary incontinence” OR “female incontinence”. The articles that included the terms “pelvic organ prolapse” OR “cancer” OR “pregnancy” were excluded. There were 780 articles in total, and it was selected only those that reported clinical trials or review articles (guidelines, meta-analysis, and theoretical or systematic reviews) involving human females not related to pregnancy or post-partum, written in English, French or Portuguese. Two hundred articles were included (49 clinical trials and 151 review articles) related to the objective of the present author. The references of recent published meta-analysis or guidelines prior to publication were excluded, since they exhibited the same evidence level of the compiled article.

### Definitions and concepts in Urogynecology

Female urinary incontinence in adults is defined as the involuntary loss of urine, and it is classified in three types: a) stress urinary incontinence (SUI), when it occurs with the increase of abdominal pressure (cough, sneeze, physical exercise, laughing, etc.); b) Urgency urinary incontinence (UUI), concurrent or with immediately following urinary urgency; c) mixed urinary incontinence (MUI), patients with stress and urgency urinary incontinence (not necessarily concurrent) (9, 10). The most prevalent is isolate SUI (51%), followed by MUI (39%) and lastly UUI (around 10%), based on clinical interview and answers of self-applied question forms. In relation to functional urodynamic evaluation, there is an increase of prevalence of pure SUI (51-77%) and reduction of MUI (11-39%), without significant alteration of isolated UUI (10-12%) (11).

### SUI

Stress urinary incontinence occurs in the presence of an increase of intra-abdominal pressure without perception of previous micturition desire. It depends on the functional adequacy of urinary sphincter and muscular and ligament structures that support female pelvic floor. When these mechanisms fail and with increase of intra-abdominal pressure (such as laughing, weight lifting, cough, sneezing) it is observed incontinence (12). The risk factors include aging, pregnancy, vaginal delivery and obesity (13). Some others are also considered, but with discrepant research results among authors: hysterectomy, diabetes mellitus, hypoestrogenism associated to menopause (11).

Incontinence grading is hard to perform, and it is admitted that severity is directly related to amount of lost urine (7), although discomfort does not reflect severity. Fultz (2003) evaluated women with urinary incontinence and observed that 28.8% refer moderate to severe discomfort (14). In a practical point of view, some authors suggest that grading may be estimated by the number of pads daily used or by the results of the “pad test” but with some doubts regarding accuracy (6). Conservative treatment of SUI (strengthening and reeducation of pelvic floor muscles) includes physical exercises of pelvic floor muscles-PFMT (pelvic floor muscle training)-and biofeedback and electro-stimulation techniques (15-17). Surgical treatment aims to correct functional inadequacy of urinary sphincter and urethra (injection of submucosal polymers around the sphincter, sub-urethral slings and Burch surgery) (6, 12, 17).

Success rate depends on the used method, but is around 51% to 91% (depending on the analyzing method, definition of cure and follow-up of every patient) (18).

### UUI

Urgency urinary incontinence is a symptom of overactive bladder (OBS) with great impact on quality of life (physical, social, psychologic and sexual aspects) (19). OAB syndrome is characterized by pollakiuria and nocturia in the absence of urinary infection or other conditions that cause the symptom. It may be associated with neurological lesions or idiopathic. Many studies on the prevalence of OAB syndrome involve individuals that seek treatment of urinary incontinence, excluding those with urgency without incontinence. In 2014 a meta-analysis was published about the epidemiology of UUI and the authors concluded that prevalence in female patients was 1% to 14%, increasing with age and directly related to socio-economic status of the population of the studied country (19).

Diagnosis of OAB syndrome is assumed in the presence of urgency urinary symptoms after exclusion of other causative causes. Flow-pressure study may demonstrate hyperactivity of detrusor (not obligatorily), what justifies the symptoms of urgency of the syndrome.

There are no sufficient data for the complete clarification of the physiologic mechanism that generates detrusor hyperactivity, felt as urgency. Except for neurologic lesions (that may provoke involuntary contractions of bladder without superior inhibition), there are four mechanisms accepted that explain detrusor hyperactivity in idiopathic OAB syndrome:

Alteration of reflex mechanism in micturition;

Reduction of innervation of muscular layer of bladder;

Release of acetylcholine in parasympathetic neuronal plaque during bladder feeling with afferent activation of bladder smooth muscle;

Activation of urothelial receptors (mucosal layer that coats bladder) (20).

The objective of OAB syndrome treatment is to eliminate symptoms, although that might be not possible..

Hyperactive bladder syndrome treatment is primarily conservative and includes teaching maneuvers/methods to inhibit involuntary detrusor contractions and strengthening of muscular structures of pelvic floor with directed exercises-PFMT. Pharmacological treatment may also be considered (antimuscarinic and ß3-agonists) for symptoms control. Other lines of treatment include intra-detrusor injection of botulin toxin neuro-stimulation (vaginal electro-stimulation, anterior tibial electro-stimulation or neuromodulation) and exceptionally bladder augmentation enterocystoplasty (5, 6, 16, 17, 20, 21).

### Mixed Urinary Incontinence

MUI patients refer incontinence associated with increase of intra-abdominal pressure and also loss or urine prior or simultaneously of urgency without abdominal effort. Patients report differently the proportion of urinary loss according to physical exercise or urgency (22). Diagnosis is based on symptoms and context when they occur. Urodynamic evaluation has little value on diagnosis, since detrusor hyperactivity is identified in only 8% of patients with urgency in the context of MUI (23). It is important to stablish which symptom is the most important (22).

The pathologic mechanisms that generate symptoms of SUI and UUI in the same patient are unknown (23).

MUI treatment aims to treat the main symptoms and the first line of treatment is the conservative recommended PFMT (6). Although there are no randomized studies of the treatment of this pathology, guidelines suggest the treatment of the main component, with a success rate lower than those observed in the treatment of isolated SUI and UUI (22).

### Alteration of quality of life (QoL) in patients with urinary incontinence

Most papers of the 80’s and 90’s discuss conservative and surgical techniques for urinary incontinence and success is defined by objective evaluation of incontinence following each intervention. In the last 10-15 years researches decided to evaluate the impact of urinary incontinence on women’s quality of life and success was related to subjective perception of each woman following intervention. In the present, it is recommended to use validated self-applied questionnaires along with physical and auxiliary exams to evaluate several domains of quality of life of women (6, 24). Urinary incontinence is not a life-threating disease, but has a highly negative impact on women’s health, affecting several aspects of daily life and quality of life, including personal, work and leisure activities (25).

In 2015, Paul Abrams published an article that evaluated the interference of urinary incontinence in quality of life of female population using an electronic question form applied to 1.203 women with urinary incontinence, with 45 to 60 years old living in USA, France, Germany and United Kingdom. He verified that incontinence grade (number of losses daily) correlated positively with interference in daily life (on daily tasks, social activities and perception of mental health well-being). The authors stressed the relationship of incontinence severity with impairment of social life, ability to visit friends and impact in familiar life (26). This work is different of previous since it addressed a younger population with intense work, familiar and social life. The results indicated that incontinence is associated with a profound sense of humiliation and stigma (25, 27-29).

The impact on quality of life is transversal to several age groups (30) and the main conditional factors are severity and type of incontinence. All incontinence types are associated to low self-esteem, and higher probability of psychiatric disease (25). The studies show higher levels of anxiety and psychological stress in women with UUI than with SUI, due to unpredictability of detrusor contractions in UUI. Asoglu (2014) verified that women with MUI or UUI had more probability to present anxiety and worse quality of life indexes than those with isolated SUI (1). Same results were obtained by a Portuguese population study by Claudia Senra (31).

The search for care due to incontinence reflects the interference on quality of life and there is a relationship of search for treatment, severity of incontinence and patient’s age (32, 33). It is speculated that the reduced search of treatment and high prevalence of incontinence may be related to shame associated (27, 34, 35). Siddiqui (2014) evaluated perceptions of incontinence and their relation to cultural and demographic aspects and concluded that urinary incontinence intimacy is similar among women of different socio-cultural status, developing adaptation strategies such as preventive micturition, identification of bathrooms in public spaces, selection of adequate outfit to eventual loss of urine and restriction of activities that are knowingly associated with incontinence. Globally they verified that women and also health professionals did not value symptoms when they were light. In relation to incontinence experience, the authors evaluated fear, stigma and shame associated, and verified a transversal negative appreciation of urinary incontinence and higher sensation of guilt in non-white population. Religious aspects (Muslims) and cultural aspects (Hispanics) showed a more negative vision of the disease than among other women (27).

### Quality of life (QoL) and the treatment of urinary incontinence patients

Globally, the treatment of urinary incontinence involves: 1) PFMT, 2) surgery and 3) pharmaceutic treatment. First line treatment usually is conservative, using exercises for the pelvic floor muscles (PFMT). This recommendation is based on an important meta-analysis using Cochrane Database in 2014 that identified its benefit in all kinds of female urinary incontinence (36). In the same year it was published a prospective article that confirmed the benefits of PFMT in quality of life of patients with urinary incontinence (37).

Surgical treatment of urinary incontinence is mainly directed to women with stress urinary incontinence or with predominance of stress leakage symptoms in mixed incontinence patients. In these cases, it is observed an improvement of quality of life based on the results of self-administered questionnaires regarding urinary symptoms and correlated disturbance of sexual life, that may not suffer the same positive impact (38).

Regarding pharmacologic treatment of urinary incontinence, the studies are mainly directed to patients with urgency incontinence. This a diagnosis based on symptoms and repercussion on quality of life and it is fundamental to determine therapeutic benefits following treatment, using drugs only in patients without cognitive disturbances (5, 6). In spite of the fact that UUI is included in overactive bladder syndrome, the use of drugs is primarily directed to reduce loss of urine episodes (5).

### Female sexual dysfunction in female urinary incontinence

The study of female sexuality is very different from the study of male sexuality. Scientific investigation regarding women doesn’t receive the same attention than that of male sexual function. Some functional studies destined to men are adapted to women and it is observed an under-valorization of sexual component in relation to reproductive female function. There is a huge gap between knowledge regarding reproductive female function and of female sexual function and its disturbances (39).

### Female sexual response

Master and Johnson at the 60’s and 70’s concluded that sexual response occurrs in a linear and sequential fashion involving four phases: arousal, plateau, orgasm and resolution. In the end of the 70’s Helen Kaplan proposed a modification of the sexual response model, reinforcing the role of sexual desire and grouping it in three phases: desire, arousal and orgasm (40). The use of this model of linear sexual response was successively questioned in relation to its applicability in women: many do not present this sexual response, and are considered inadequate, even if they do not considered themselves as such. The sequence of phases was also questioned, since there was an overvaluation of biological and physical phenomena in detriment of conditionings of female sexual pleasure and satisfaction (41). Basson (2001) presented a non-linear model of female sexual response integrating emotional, psychological, and cognitive aspects and external sexual stimulants. According to this model, sexual response starts with sexual desire (spontaneous or not, externally or through cognitive motivation). Sexual arousal includes subjective sensation of arousal or physiological arousal with poor correlation between them (40). The presence of sexual desire and sexual arousal are not sequential although both are important to achieve sexual satisfaction (41). This model is adopted by International Consensus on Sexual Medicine, International Classification of Diseases (ICD-10) and also by the diagnostic and statistical manual of mental diseases (DSM-5) (40).

### Female sexual dysfunction and evaluation methods

Prevalence studies on female sexual dysfunction estimate the existence of sexual disturbances in 39-45% of sexually active women (40, 42, 43). This evaluation must be balanced since the studies did not match diagnostic criteria with the data in which the studies were realized (for example, DSM) and symptoms grading. International accepted criteria are present only for the diagnosis of dysfunctions and this is considered when are excluded: 1) Mental diseases not related to sexual function; 2) iatrogenic pharmacological treatment or clinical condition that justifies symptoms; 3) severe disturbance of relationship, 4) violence of partner or 5) other stressing factors (44). Sexual disturbances associated to incontinence cannot be classified as sexual dysfunction according do DSM-5 classification, even with personal suffering and prejudice to sexuality.

The presence of urinary symptoms in incontinent women justifies an adequate gynecological exam and evaluation of sexual function (42, 45). It is also recommended the use of self-administered questionnaires with good diagnostic sensitivity (40). European Urological Association guidelines of diagnosis and therapeutics of urinary incontinence recommend the use of the following question forms to evaluate sexual function in the presence of urinary symptoms: FSFI (Female Sexual Function Index), ICIQ-VS (International Consultation on Incontinence Questionnaire-Vaginal Symptoms), PISQ-IR (Pelvic Organ Prolapse/Urinary Incontinence Sexual Questionnaire-IUGA revised), SQoL-F (Sexual Quality of Life-Female) and SFQ (Sexual Function Questionnaire) (6, 24). All of them have been used in patients with SUI, UUI and MUI (6). FSFI (46) and ICIQ-VS (47) as well as the earlier version of PISQ-IR (PISQ-12 (48) are validated in Portuguese.

### Sexuality and urinary incontinence

Although it is assumed that there is a high probability of influence of urinary incontinence on sexual life, the studies present very different results probably due to the great variability of investigation methods (12). Urinary incontinence may trigger problems related to sexual female life, namely: loss of urine during coitus (coitus incontinence), night losses associated to urgency and fear of bedwetting (49-51). Fear of malodorous and urinary incontinence during coitus are associated with alteration of image and self-esteem responsible for low frequency of sexual activity among incontinent women (52). In elderly population, in the presence of a sexual partner, the occurrence of urinary incontinence has a negative impact on sexuality (8).

Urinary incontinence related to coitus has been described in two ways: urinary incontinence associated to penetration and associated to orgasm (“squirting”) (53). Incontinence associated to penetration was associated to SUI and is related to probable intrinsic dysfunction of sphincter, and it is more frequent in women with SUI demonstrated by urodynamic evaluation (18). Coitus urinary incontinence associated to orgasm has been related to detrusor overactivity, although data are not fully clarified (54-58).

Several papers studied the relationship of different kinds of urinary incontinence and sexuality. Asoglu (2014) concluded that urgency symptoms, especially in the presence of MUI, were associated to anxiety disturbances, mood disturbances (depression symptoms) and low quality of life of SUI in the context of sexual life (1). Su (2015) evaluated sexual function in the presence of urinary incontinence, using international validated questionnaires (FSFI) and identified differences among different domains on sexual function according to different types of urinary incontinence. UUI related to reduction to lubrication and increase of pain associated to sexual activity. MUI was related to reduction of sexual satisfaction while SUI did not present any impact on sexual relation (59).

### Sexuality after urinary incontinence treatment

Conservative treatment of urinary incontinence using PFMT presented an improvement of functional parameters of the domains desire, arousal and orgasm, regardless the type of urinary incontinence (3). This benefit is mainly relevant in patients with an initial evaluation with significant disturbances of sexual function (59). The studies point an improvement of sexual function with the strengthening of pelvic floor muscles in SUI, including patients with coitus incontinence associated to penetration (1, 2, 60-62).

Surgical treatment of urinary incontinence has been studied in the context of SUI treatment. Published revisions of 2012 (52), 2014 (12), and 2015 (63) highlight the difficulty of evaluation of this parameter due to the absence of uniformization of methodology but recognize the global concern of evaluation of sexual life. It is cited an improvement of sexual function in patients with SUI that also presented coitus urinary incontinence. Women without incontinence during coitus prior to intervention did not present improvement of sexual function even in the presence of improvement of global quality of life. In a percentage of patients submitted to surgical correction of SUI using suburethral slings there is prejudice of sexual function, including reduction of libido, dyspareunia or sexual inactivity. It seems that this report is associated to failure of surgical intervention (12).

Surgical treatment of UUI (through sacral neuromodulation) is poorly studied in relation to sexual function and the existent studies involve small samples of patients but in general show improvement of questionnaire evaluation of sexual function (63-65). In 2015, a meta-analysis was published that considered that there is insufficient data to conclude the impact of sacral neuromodulation on female sexuality (66).

First line of treatment of clinical treatment of OAB syndrome and UUI includes anti-muscarinic and beta3-agonists. There are very few studies about the repercussion of those drugs on sexual function. Only studies of oxibutinin and tolterodin have been published on the subject. Oxibutinin lowered coitus incontinence, associated shame/disturbance and improved sexual life, relationship with partner and increase of sexual interest. Tolterodin showed a higher sexual and mental health based on questionnaires SQoL-F and PISQ-IR and improvement of anxiety scales of evaluation (67). There are no published data about the use of mirabegron (beta3-agonist) on female sexual function.

## CONCLUSIONS

The presence of urinary incontinence is associated to stigma, fear, embarrassment and shame related to clinical condition, with repercussion on self-esteem and disturbance of personal, social and sexual life.

Urinary incontinence affects negatively female sexual life. Fear of intimacy associated to sex activity is evident in view of the lower frequency of sexual activity and low sexual and global satisfaction indices among incontinent women. The development of adaptation strategies to incontinence may reduce the impact that the loss of urine may have on sexual activity, but these techniques have greater benefit on stress urinary incontinence.

Conservative treatment (PFMT) of urinary incontinence improves quality of life and sexuality, regardless the type of incontinence. Studies demonstrate an improvement of quality of life and sexual function after surgical treatment of SUI; the improvement is higher if pre-operatively it is demonstrated the repercussion of incontinence on sexuality. After treatment of UUI, it is identified an improvement of quality of life and sexual function in individuals who receive anticholinergics. Sacral neuromodulation has a positive influence on both domains, but for definite conclusion more studies are necessary.

## ARTICLE INFO

Int Braz J Urol. 2017; 43: 20-8
